# The ground of ground, essence, and explanation

**DOI:** 10.1007/s11229-018-1856-y

**Published:** 2018-06-26

**Authors:** Michael Wallner

**Affiliations:** Department of Philosophy, University of Graz, Heinrichstraße 26, 8010 Graz, Austria

**Keywords:** Metaphysics, Grounding, Essence, Explanation, Fundamentality

## Abstract

This paper is about the so-called meta-grounding question, i.e. the question of what grounds grounding facts of the sort ‘*ϕ* is grounded in *Γ*’. An answer to this question is pressing since some plausible assumptions about grounding and fundamentality entail that grounding facts must be grounded. There are three different accounts on the market which each answer the meta-grounding question differently: Bennett’s and deRosset’s “Straight Forward Account” (SFA), Litland’s “Zero-Grounding Account” (ZGA), and “Grounding Essentialism” (GE). I argue that if grounding is to be regarded as metaphysical explanation (i.e. if *unionism* is true), (GE) is to be preferred over (ZGA) and (SFA) as only (GE) is compatible with a crucial consequence of the thought that grounding *is* metaphysical explanation. In this manner the paper contributes not only to discussions about the ground of ground but also to the ongoing debate concerning the relationship between ground, essence, and explanation.

## Introduction

This paper is about the so-called meta-grounding question, i.e. the question of what grounds grounding facts of the form ‘*ϕ* is grounded in *Γ*’.^1^ An answer to this question is pressing since some plausible assumptions about grounding and fundamentality entail that grounding facts must be grounded. Three different accounts have been proposed to answer the meta-grounding question: Bennett (2011) and deRosset (2013) independently develop accounts according to which grounding claims like ‘*ϕ* is grounded in *Γ*’ are themselves grounded in *Γ*. Call this the “Straight Forward Account” (SFA).^2^ Litland’s (2017) “Zero-Grounding Account” (ZGA) has it that grounding claims are *zero*-*grounded*, i.e. grounded in the *empty set of facts*. And finally, according to a position that I call “Grounding Essentialism” (GE), grounding facts like ‘*ϕ* is grounded in *Γ*’ are grounded in essentialist facts, i.e. in facts about the essences of *ϕ*, or *Γ*, or both. Versions of (GE) are discussed in Rosen (2010), Fine (2012) and Dasgupta (2014).^3^

I am going to investigate the implications of these accounts concerning the relation between grounding and metaphysical explanation. While some grounding theorists—call them *unionists* (Raven 2015)—identify grounding with metaphysical explanation, others—call them *separatists* (Raven 2015)—maintain that grounding and metaphysical explanation come apart. On the latter view, however, grounding is still an explanatory notion in as much as it *backs* metaphysical explanation, much like causation backs causal explanation (Schaffer 2016). I argue that (ZGA) and (SFA) are incompatible with unionism. I will show that unionist versions of both (ZGA) and (SFA) clash with a crucial intuition about metaphysical explanation. This amounts to a criticism of Litland’s (2017) account, since he endorses both unionism and (ZGA). It can also be taken as a criticism of deRosset (2013), in as much as he endorses a unionist version of (SFA).^4^ The arguments in this paper show that *if* grounding is metaphysical explanation (i.e. if *unionism* is true), (GE) has the edge over its competitors (SFA) and (ZGA). The main goal, however, is not to argue for a specific answer to the meta-grounding question, nor to decide the debate between unionism and separatism. The main thesis the paper seeks to establish is that the correct account of what grounds grounding at least partly depends on the answer to the question of whether unionism or separatism is true.

Here is an overview of the paper: I will start with some very brief remarks about unionism and separatism in Sect. 2 and about the relation between grounding and fundamentality in Sect. 3. I then develop the critique of Litland’s unionist version of (ZGA) in Sects. 4–6. In Sect. 7, I outline the general idea of (GE). I address the relation between (GE) and unionism in Sect. 8. I then sketch (SFA) in Sect. 9 and argue that it is incompatible with unionism in Sect. 10. Finally, Sect. 11 concludes.

## Grounding and explanation

The notion of grounding is much discussed in contemporary metaphysics. Grounding theorists hold that grounding is intimately tied to two connected notions: *metaphysical explanation* and *fundamentality*. I will briefly discuss grounding’s connection to these notions in turn. Grounding is often captured by idioms like “holding in virtue of” or by specific *non*-*causal* uses of “because”. There is a widespread agreement amongst grounding theorists that grounding is an *explanatory notion*. Yet, there are two ways in which this idea can be cashed out. Some theorists take grounding simply to be metaphysical explanation, such that “to say that *ϕ*_*1*_, *ϕ*_*2*_, … ground *ϕ* just is to say that *ϕ*_*1*_, *ϕ*_*2*_, … explain *ϕ* in a distinctively metaphysical way” (Litland 2017, p. 281). Raven (2015) calls this position *unionism*. Other proponents of grounding hold that grounding and metaphysical explanation come apart. On this view, grounding proper is not itself metaphysical explanation but *backs* metaphysical explanation. Raven (2015) calls this position *separatism*.

Separatists like Rodriguez-Pereyra (2005), Audi (2012a, b), Schaffer (2012) and Trogdon (2013) have different reasons to argue for separatism. Audi (2012a, p. 119) sees separatism as the best response to the following objection: The attempt to explicate grounding by saying ‘*x* (at least partly) *metaphysically explains y* just in case *x* grounds *y*’ would amount to mere relabeling if grounding just were metaphysical explanation. Schaffer (2012, p. 124) refers to similarities in the debate about causation and causal explanations and argues that grounding and metaphysical explanation should be kept apart in the same way that causation and causal explanation have to be distinguished. Schaffer also argues for separatism as he wants objects to figure in grounding claims but realizes that they are inapt to figure in explanations.^5^,^6^

Among the unionists we find Rosen (2010), Fine (2012), Raven (2012), deRosset (2013), Dasgupta (2014) and Litland (2017). One motivation for defending unionism can be found in the complexity of the separatist thesis. Note that the separatist (as opposed to the unionist) is left with three tasks instead of just one. She has to give an account of grounding and, as they come apart, of metaphysical explanation, while in addition she has to account for their connection—the backing relation. In this paper I am *not* going to argue for either of these positions. I will, however, argue that the way we decide this dispute has impact on the way we are able to answer the question of what grounds grounding claims.

## Grounding, fundamentality, and the ground of ground

Another tight connection that has been pointed out in the literature is that between grounding and fundamentality. Grounding is said to depict the *layered structure of reality*. By stating what grounds what, we are displaying the world’s layered structure from the fundamental to the increasingly derivative. That is to say grounding is intimately linked to fundamentality, such that the following principle (F) should hold:

(F): A fact *f* is fundamental iff it is ungrounded; equivalently, *f* is non-fundamental or derivative iff it is grounded in some other fact(s).^7^

Against this backdrop, the question of whether or not grounding facts are *themselves grounded* becomes the question of whether or not grounding facts are *fundamental*. Given a very plausible assumption, it is easy to see that grounding facts cannot be fundamental. This is Sider’s (2011, p. 106) formulation of the assumption in question:

(Purity): Fundamental truths involve only fundamental notions.

A fundamental notion is a notion that carves reality at its joints. By this standard, the *notion of being a city* is not fundamental, according to Sider. Consider the following truth:

1. There exists a city.

By (Purity), (1) is not a fundamental truth, for it involves a non-fundamental notion. Thus, by (F), (1) has to be grounded.

(2) That there is a city *is grounded in T*.

The question of whether or not (2) is itself grounded, according to (F), is tantamount to the question of whether or not (2) is fundamental. If (Purity) is true, however, (2) cannot be fundamental, since (2) contains the non-fundamental notion of being a city. If (F) is true, then this means that grounding facts like (2) are themselves grounded.^8^

This is what makes the so-called meta-grounding question, the question of *what* grounds grounding facts, so pressing. In the light of (F) and (Purity), grounding facts like (2) must themselves be grounded.^9^,^10^ We now need to ask *what* it is that grounds grounding facts.

## Litland’s Zero-Grounding Account (ZGA)

Litland (2017) argues that grounding claims are *zero*-*grounded*. This, as we shall see shortly, means that they are grounded in the empty set of facts or statements. Central to Litland’s approach to iterated ground is the distinction between factive and non-factive ground. While most philosophers think that grounding is factive, it is easy to derive from a factive notion of grounding a non-factive version, according to which also falsehoods may flank the grounding connective. Litland, however, takes non-factive ground as the primitive notion from which a factive version of ground can be derived. He uses the already familiar sentential operator ‘<’ for factive grounding statements and the sentential operator ‘⇒’ for non-factive grounding statements.^11^ With the distinction between factive and non-factive ground in hand, we can sketch Litland’s (ZGA):

(ZGA): If *Γ* < *ϕ*, then *Γ* < *ϕ* is grounded in (i) *Γ* and (ii) *Γ* ⇒ *ϕ.*
  *Γ* ⇒ *ϕ* is *zero*-*grounded*.

Every true, non-factive grounding claim *Γ* ⇒ *ϕ* is (factively) grounded in the empty collection of statements. Borrowing from Fine (2012), Litland expresses this by saying that every true, non-factive grounding claim is *zero*-*grounded*. And since every factive grounding claim is partially grounded in the corresponding non-factive grounding claim, every factive grounding claim is partially, mediately grounded in the empty collection of statements. In addition, it follows from (ZGA) that if some truth *ϕ* is (factively) zero-grounded, then the truth that *ϕ* is (factively) zero-grounded (0 < *ϕ*) is itself (factively) zero-grounded (0 < (0 < *ϕ*)).^12^,^13^

In order for us to be able to evaluate (ZGA), Litland needs to provide answers to the questions of (a) what exactly is meant by zero-grounding and of (b) why we should believe that all true, non-factive grounding claims are zero-grounded.^14^ Litland follows Fine (2012, p. 47) in distinguishing cases in which a statement is *un*grounded from cases in which a statement is *zero*-grounded. In the former case, the statement is not grounded at all—no number of sentences serve as ground for it—whereas in the latter case the fact is grounded, there is a number of sentences in which it is grounded and that number is zero. The distinction between ungrounded and zero-grounded facts is to be understood analogously to the familiar distinction between propositions that are not derivable and propositions that can be derived from the empty collection of premises (Litland 2017, p. 280).

Litland’s official story about zero-grounding is told within the framework of “*explanatory arguments*”.^15^ This notion is best understood in contrast to what Litland calls “plain arguments”.

(PA): If there is a *plain argument* from premises Δ to conclusion *ϕ*, then if each δ ∈ Δ is true, *ϕ*, too, is true (Litland 2017, p. 290).
(EA): If there is an *explanatory argument* from premises Δ to conclusion *ϕ* then, if each δ ∈ Δ is the case, then this fully explains its being the case that *ϕ* (Litland 2017, p. 290).

Put differently, explanatory arguments are solely composed from *explanatory inferences*. Litland links the notion of explanatory inferences to immediate ground in the following way:

(EI): If *Γ* immediately grounds *ϕ*, the inference from *Γ* to *ϕ* is explanatory (Litland 2017, p. 289).^16^

After having further elucidated the notion of explanatory arguments, Litland establishes the following link between grounding and explanatory arguments:

(GEA): Δ < *ϕ* iff (i) each δ ∈ Δ is the case and (ii) there is an explanatory argument from Δ to *ϕ* (Litland 2017, p. 293).

From (GEA) one can derive a link between *non*-factive grounding and explanatory arguments by dropping the factivity condition (i):

(GEA_NF_): Δ ⇒ *ϕ* iff there is an explanatory argument from Δ to *ϕ*.^17^

With this link in hand, we can now interpret ‘Δ ⇒ *ϕ*’ as referring to the fact that there is an explanatory argument from Δ to *ϕ*. How does this help us to elucidate what zero-grounding is? In light of the link between grounding and explanatory arguments a statement is zero-grounded iff there is an *explanatory argument* to this statement from the *empty set of premises*.^18^ How do explanatory arguments help us to motivate the claim that all non-factive grounding claims indeed are zero-grounded? Litland develops a pure logic of iterated ground by suggesting introduction- and elimination-rules for grounding operators on the basis of their link to explanatory arguments. This allows him to formally prove that all non-factive grounding claims are zero-grounded. Due to reasons of space I cannot discuss Litland’s logic and his proof. Instead of detailing his formal argument that all true, non-factive grounding claims are zero-grounded for (ZGA), I would like to further examine the *consequences* of (ZGA). For this purpose it is not necessary to discuss the formal details of Litland’s proof. It will suffice to have clarified what it means that some *ϕ* is zero-grounded—it means that there is an explanatory argument to *ϕ* from the empty set of premises.

## (ZGA) and unionism are incompatible

Litland discusses an objection to his (ZGA) that I take to be very revealing. It is a consequence of his account that every true, non-factive grounding claim has the same ground—the zero-ground. Thus, on Litland’s unionist approach to grounding, this means that every true, non-factive grounding claim has the same explanation. But intuitively, the explanation for why *p* ⇒ *p *∧ *p* is the case differs from the explanation for why *p* ⇒ *p *∨ *p* is the case (Litland 2017, pp. 302–303).^19^ The former explanation should have to do with what *conjunction* is, the latter with what *disjunction* is. Note that, according to (ZGA), the fact that *p* ⇒ *p *∧ *p* is zero-grounded amounts to the fact that there is an *explanatory argument* from the *empty set of premises* to *p* ⇒ *p *∧ *p*. According to the objection, however, it is plausible that some fact about conjunction figures in the *explanans* of *p* ⇒ *p *∧ *p* and, hence, that it is not the empty set of premises (i.e. not the zero-ground) *alone* that *explains* why *p* ⇒ *p *∧ *p* holds. To further illustrate the plausibility of this, we can look at a different example of an argument from the empty set of premises. The trivial truth that I can’t eat that cake and simultaneously not eat it follows from the empty set of premises by the law of non-contradiction. There is an argument from the empty set of premises to the trivial cake-truth. Whether this argument is *explanatory* hinges on whether the *inferences* involved are explanatory. Let’s suppose they are (Litland (2017, p. 290) does not wish to ultimately settle which inferences are and which are not explanatory). If the argument from the empty set of premises to the trivial cake-truth is explanatory, then on (ZGA) we can say that the trivial cake-truth is zero-grounded. Yet, intuitively, it is not the empty set of premises, the zero-ground, by itself that *explains* why the trivial cake-truth holds. It is plausible that any successful explanation of why the trivial cake-truth holds will include the law of non-contradiction among the *explanantia*.^20^

This objection casts doubt on a *unionist version* of (ZGA). If it is plausible that *p* ⇒ *p *∧ *p* and *p* ⇒ *p *∨ *p* have different metaphysical explanations, then (ZGA) must hold that grounding and metaphysical explanation come apart, since the central claim of (ZGA) is that all grounding facts have the same ground—namely the zero-ground. But unionism holds that grounding just *is* metaphysical explanation. Conclusion: if the two grounding claims have different explanations, then (ZGA) must reject unionism. Put differently, the following triad is inconsistent:

(DE): *p* ⇒ *p *∧ *p* and *p* ⇒ *p *∨ *p* have different metaphysical explanations.
(ZGA): All true, non-factive grounding claims are zero-grounded.
(U): Grounding is metaphysical explanation.

For Litland to maintain his unionist version of (ZGA) he has to reject (DE). So, what is it that Litland offers as a response to the objection based on (DE)? Litland argues that the objection is misguided, since it falls prey to an ambiguity in what is meant by “explanation”:

In the framework of the explanatory arguments we can see that the objection trades on an ambiguity in what is meant by “explanation”. In this framework there are two things one can mean by an explanation of *ϕ*: one can mean a collection of propositions *ϕ*_*1*_, *ϕ*_*2*_, … from which *ϕ* can be derived in an explanatory way; alternatively, one can mean an *argument* witnessing that *ϕ* can be derived in an explanatory way. (Litland 2017, p. 303)

While *p* ⇒ *p *∧ *p* and *p* ⇒ *p *∨ *p* have the same explanation in the first sense (in both cases there is an explanatory argument to each of these grounding claims *from the empty set of premises*), *p* ⇒ *p *∧ *p* and *p* ⇒ *p *∨ *p* do not have the same explanation in the second sense. This is because the explanatory *arguments* that witness that these two grounding claims can be derived from the empty set of premises are different in the two cases. What makes them different is that they employ different inference-rules (Litland 2017, p. 303).^21^ It is in virtue of the different inference-rules that the explanations of the two grounding claims differ in the second sense of explanation.

Hence, I take it that in this second sense of explanation the *rules* by which the *explanandum* follows from the premise-set have to figure in the *explanantia* of the grounding facts. So, in the framework of explanatory arguments, the crucial difference between Litland’s two senses of explanation concerns what figures in the *explanantia* of grounding facts. While in the first sense of explanation it’s *only* the premise-set that figures in the *explanans* of the grounding fact, in the second sense it is *also* the explanatory inference rules on the basis of which the respective grounding claim follows from the empty set of premises.

It is helpful to have some names to refer to these different kinds of explanations. I propose the following terminology: I will speak of the latter kind of explanation as a ‘rule-explanation’, and I will speak of the former kind of explanation as an ‘input-explanation’, since the premise-set can be understood as the input to an explanatory argument in the former kind of explanation. Note that the name ‘*rule*-explanation’ should not suggest that it’s *only* the rules that do the explanatory work in the second sense of explanation. Yet, calling it ‘*rule*-explanation’ signals the distinctive feature that—contrary to input-explanations—the rules also figure in the *explanantia* of grounding facts.

In this terminology Litland’s response to the objection is that (DE) is only plausible if grounding claims have rule-explanations instead of input-explanations. (ZGA) offers an *input*-explanation for grounding claims and on this picture it is plain that it is the zero-ground that *input*-explains all true, non-factive grounding claims. It is also plausible that, on the next iteration, the zero-ground *input*-explains the existence of an explanatory argument from the *empty set of premises* to *p* ⇒ *p *∧ *p*, and so on.

By itself, this response does not appear sufficient. After all, the objector is likely to reply that the plausibility of (DE) renders any type of explanation that assigns the same *explanans to p* ⇒ *p *∧ *p* and *p* ⇒ *p *∨ *p* unsatisfactory. Hence, from the objector’s point of view, Litland cannot cast doubt on the plausibility of (DE) merely by assuming or introducing another kind of explanation, input-explanation, for which (DE) is not plausible. However, Litland also tries to explain away the plausibility of (DE) by offering a different picture, according to which (DE) is false but something in the vicinity of (DE) is true. He distinguishes between *different ways* in which facts can be zero-grounded. “[W]hile every true non-factive grounding claim Δ ⇒ *ϕ* has the same (immediate) strict full ground—the empty ground—different true non-factive grounding claims are (immediately) zero-grounded in different *ways*.”^22^ (Litland 2017, p. 303) The *ways* in which different grounding claims are zero-grounded correspond to the different inference-rules involved in the respective explanatory argument from the empty set of premises to the respective grounding claim. The explanatory argument from the empty set of premises to *p* ⇒ *p *∧ *p* uses different inference-*rules* than the argument from the empty set of premises to *p* ⇒ *p *∨ *p*. According to (ZGA), these two grounding claims, *p* ⇒ *p *∧ *p* and *p* ⇒ *p *∨ *p*, are indeed both zero-grounded, yet *in a different way*. Given unionism, this means that the two grounding claims are indeed both *explained* by the appeal to the zero-ground, yet each *in a different way*.

It is doubtful, however, whether Litland’s alternative picture about the different *ways* in which *p* ⇒ *p *∧ *p* and *p* ⇒ *p *∨ *p* are explained by the same *explanans* suffices to reduce the plausibility of (DE). Note that (DE) is intuitively supported by considering particular examples of grounding claims, namely *p* ⇒ *p *∧ *p* and *p* ⇒ *p *∨ *p*. Additionally, the example revolving around the trivial cake-truth provides further reason to think that it is unsatisfactory to only appeal to the zero-ground in metaphysically explaining grounding facts. On the other hand, consider Litland’s picture about grounding facts having the *same explanation* but differing in the *ways* in which they are explained. Litland only offers one reason to accept this picture: its consistency with (ZGA). However, consistency with (ZGA) can also be maintained by dropping unionism and keeping (DE). Therefore, Litland is in a dialectically difficult position: (DE) is intuitively supported and can be preserved by abandoning the conjunction of (ZGA) and unionism, while the alternative picture Litland offers does not enjoy comparable intuitive support.

This is not meant to be a knockdown argument against the compatibility of (ZGA) and unionism. The above considerations only show that Litland’s defense leaves him in a dialectically weak position. However, it is also possible to argue directly for the incompatibility of (ZGA) and unionism. I will develop this argument in the rest of this section.

The success of a unionist version of (ZGA) is intimately tied to the question whether appealing to the zero-ground alone can be seen as a satisfactory metaphysical explanation of a grounding claim like, say, *p* ⇒ *p *∧ *p*. To decide on this question, we have to first think about what constitutes a satisfactory metaphysical explanation of *p* ⇒ *p *∧ *p*. In light of the framework of explanatory arguments, it is the following question (Q) that a metaphysical explanation of *p* ⇒ *p *∧ *p* has to answer:

(Q): Why is there an explanatory argument from *p* to *p *∧ *p*?

Note that it is of utmost importance to Litland’s framework of explanatory arguments that if an answer to this question is to be a metaphysical explanation of a grounding claim, *p* ⇒ *p *∧ *p*, the argument from *p* to *p *∧ *p* needs to be an explanatory one. This is because mere plain (or non-explanatory) arguments do not account for a grounding relation between the premise-set and the conclusion. Hence, answering (Q) requires showing why the argument from *p* to *p *∧ *p* is explanatory. Another way to put this is that grounding claims express an explanatory connection between the ground and the grounded and not just any connection.^23^ So, what we seek in a metaphysical explanation of a grounding fact is an explanation of why the connection between the ground and the grounded is explanatory. I, thus, conclude that any satisfactory answer to (Q) must tell us *why* the argument from *p* to *p *∧ *p* is explanatory.

I will now show that merely appealing to the zero-ground cannot tell us why the argument from *p* to *p *∧ *p* is explanatory and that, therefore, (ZGA) is ill fitted to answer (Q): Consider the argument from *q* to *p *→ *p.*^24^ There surely is *some such argument*. Yet, *q* does not ground *p *→ *p*, factively or otherwise. This is because this argument is *not explanatory*.^25^ (ZGA) predicts the right results here. Still, the argument from *q* to *p *→ *p* is *valid*. This means that the fact that there is an argument from *q* to *p *→ *p* can be derived from the empty set of premises: If we need no additional premise (other than *q*) to conclude *p *→ *p*, it is safe to say that we do not need any premises to conclude that we can conclude *p *→ *p* from *q*. Analogously, we can derive the fact that there is an argument from *p* to *p *∧ *p* from the empty set of premises. It seems that both, the fact that there is an argument from *q* to *p *→ *p* as well as the fact that there is an argument from *p* to *p *∧ *p*, can be accounted for (or: *input*-explained) by appealing to the empty set of premises. This means that the appeal to the empty set of premises does the same explanatory work in both cases. The crucial *difference* between the two arguments in question, however, is that the latter is explanatory, while the former is not. Yet, since the mere appeal to the empty set of premises, the zero-ground, does the same explanatory work in both cases, merely appealing to the zero-ground cannot explain the crucial difference between those cases. Therefore, (ZGA)’s claim that *p* ⇒ *p *∧ *p* is zero-grounded does not explain *why* the argument from *p* to *p *∧ *p* is *explanatory*. So, (ZGA) cannot answer (Q). But, as I have argued, an account of the ground of grounding facts *should* answer (Q), *if we assume unionism*. I conclude that (ZGA) and unionism are incompatible.

It is important to note that, even though appealing to the empty set of premises does the same explanatory work in the case of the *explanatory* argument as it does in the case of the *non*-*explanatory* argument, this does *not* entail that the zero-ground bears the same *connection* to both cases. Quite the opposite is the case: The empty set of premises relates to the fact that there is an argument from *p* to *p *∧ *p* via *explanatory* inference rules, while it relates to the fact that there is an argument from *q* to *p *→ *p* via *non*-*explanatory* inference rules. This indicates that it is really the different inference-rules in play that account for whether the argument in question is an explanatory one. So, it is the appeal to inference-rules rather than to the zero-ground that answers (Q).

Note that this is supported by Litland’s own characterization of explanatory arguments. Litland (2017, p. 289) emphasizes that it’s the inferences that make an argument explanatory. In his examples of explanatory inferences Litland talks about inference *rules* like conjunction-introduction or disjunction-introduction.^26^ This suggests that arguments are explanatory in virtue of the inference *rules* they employ. From this perspective it is clear that the mere appeal to the zero-ground does not suffice to explain why the argument in question is explanatory. These considerations much rather motivate the view that the rules involved should be part of the *explanantia* of grounding facts, i.e. the view that grounding facts should be rule-explained rather than input-explained.

By my lights, the claim that Δ ⇒ *ϕ* is zero-grounded just amounts to holding the following: We don’t need any premises to derive Δ ⇒ *ϕ* in an explanatory way *because* the inferences applied in getting from Δ to *ϕ* are *explanatory*. My point is that if it is really the inference rules that make the argument *explanatory*—which Litland (2017, p. 289) arguably concedes—it is plausible that they figure in the *explanans* of the grounding fact Δ ⇒ *ϕ* (i.e. the fact that there is an explanatory argument from Δ to *ϕ*). If this is true, however, (ZGA) can only maintain that Δ ⇒* ϕ* is zero-grounded if grounding and metaphysical explanation come apart. So, (ZGA) and unionism mutually exclude each other, and Litland cannot defend (ZGA) while simultaneously defending unionism.

It is important to note the scope of this objection. It rejects a *unionist version* of (ZGA) but remains silent about the compatibility of (ZGA) and separatism. For all I have said, there might still be an explanatory connection between the zero-ground and true, non-factive grounding claims. If my arguments are sound, however, this explanatory connection is not identical to metaphysical explanation.^27^

## Begging the question against (ZGA)?

It is now time to consider an objection to my criticism of a unionist version of (ZGA) and to the claim that the inference-rules involved in an explanatory argument from the ground to the grounded figure in a metaphysical explanation of the existence of the *explanatory argument* in question.

*Objection* The above criticism of a unionist version of (ZGA) and the argument according to which the rules play a role in the metaphysical explanation of the zero-grounded grounding fact begs the question against the proponent of (ZGA). According to (ZGA), grounding facts are themselves grounded in the empty set of facts. So, any *explanatory argument* whose conclusion is a grounding claim has zero premises, i.e. *zero explanantia*. The whole point of (ZGA) is that there can be explanatory arguments, and therefore *explananda* that really are explained, without there being any *explanans* that explains them. So, it is entirely misguided to ask, then, what explains the *explananda* of explanatory arguments that are zero-grounded (in the sense of being their *explanans*), and to propose that it’s the rules or the laws that do that. Nothing explains grounding facts in the sense of serving as their *explanantia*—not other facts, not laws, not principles, etc. Grounding facts nonetheless differ from fundamental facts, since they are explained; there’s an explanation, in the sense of an explanatory argument, that has them as its conclusion. This is not true of fundamental facts.^28^

*Response* The objection maintains that the whole point of (ZGA) is that there can be explanatory arguments, and therefore *explananda* that *really are explained*, without there being any *explanans* that explains them. I contend that in order for the *explananda* to *really be explained*, the argument in question needs to be an *explanatory* one (as opposed to a mere *plain* or non-explanatory argument). Since it is the inference-rules that make an argument explanatory (Litland 2017, p. 289), the rules are doing explanatory work.^29^ Maybe grounding facts are zero-grounded, but the zero-ground (alone) does not suffice to metaphysically explain them. The objector’s characterization of (ZGA) has it that nothing explains them, though they are explained. I argue that the objection overlooks that the argument from the ground to the grounded needs to be *explanatory* (instead of just plain or non-explanatory) (Litland 2017, p. 290). I take it that what makes the argument explanatory, the inference-rules, also does explanatory work.

Another way to make the point is the following: The claim that there is an explanation of grounding claims without there being any *explanantia* is a large bullet to bite, so much so, I think, that it would greatly decrease the plausibility of (ZGA) itself. Introducing the distinction between input- and rule-explanation, however, provides us with an *explanans* that does the explanatory work even though there is no input (i.e. no premises) in the explanatory argument from the ground to the grounded. This is because in a rule-explanation the rules at work in an explanatory argument crucially figure in the *explanantia* of grounding claims. Note that this is consistent with the claim that all true, non-factive grounding claims are zero-*grounded*, i.e. with (ZGA), yet not with a *unionist version* of (ZGA).

## Grounding essentialism (GE)

Dasgupta (2014, p. 566) offers the following example to illustrate how grounding claims work:

(C): The fact that event *e* contains people engaged in conference-conducive activities (C-activities) grounds the fact that *e* is a conference.

The question of what grounds (C) amounts to the question of *why* the fact that *e* contains people engaged in C-activities makes it the case that *e* is a conference. A natural answer seems to involve some facts about what conferences are, i.e. some facts about the *essential connection* between conferences and C-activities. It lies in the essence of conferences that an event is a conference if it involves people engaged in C-activities. According to Dasgupta (2014, pp. 566–567), this essentialist fact about conferences is also *why* the fact that *e* contains people engaged in C-activities makes it the case that *e* is a conference. Thus, on this *essentialist* view, (C) is (at least partially) grounded in the *essential connection* between (some or all constituents of) the grounded fact and (some or all constituents of) its ground. I call this view “grounding essentialism” (GE):

(GE): If *Γ* < *ϕ*, then (*Γ* < *ϕ*) is (at least partially) grounded in the *essential connection* between (some or all constituents of) *Γ* and *ϕ.*

This essential connection between (the constituents of) *Γ* and *ϕ* can be captured by an *essentialist fact* about (the constituents of) *Γ*, or *ϕ*, or both. Thus, (GE) can also be formulated the following way:

(GE′): If *Γ* < *ϕ*, then (*Γ* < *ϕ*) is (at least partially) grounded in some essentialist fact *f* about (some or all constituents of) *Γ*, or *ϕ*, or both (capturing the essential connection between (some or all constituents of) *Γ* and *ϕ*).^30^

Rosen (2010), Fine (2012) and Dasgupta (2014) all discuss versions of (GE).^31^ Their versions differ regarding the exact way in which the essential connection between the ground and that which is grounded is to be spelled out. (GE), in general, is the view that grounding facts hold at least partly in virtue of the nature of that which is grounded, or of its ground, or of both.^32^

## (GE) and unionism

In Sect. 5 I use Litland’s framework of explanatory arguments to argue for the following claim: On a unionist picture, the *meta*-grounding question, i.e. the question of why there is an *explanatory* argument from the ground to the grounded, cannot be answered without appealing to the inference-*rules* that are involved in such an argument. It is now time to determine what this means outside the framework of explanatory arguments.

It is plausible that a grounding claim like *Γ* < *ϕ* expresses the fact that *Γ* and *ϕ* are in a specific, explanatory sense metaphysically connected. A metaphysical explanation of this very fact should answer the question of *why* this connection holds. In other words, it should tell us what accounts for this metaphysical explanatory connection.^33^ Schaffer (2017a, p. 3; 2017b, pp. 308–309) argues that explanation has a *tripartite structure* of *source*, *link,* and *result*. In the case of causal explanation, we have *cause*, *laws of nature*, and *effect*. Logical explanation involves *premise*, *inference rule*, and *conclusion*. Analogously, metaphysical explanation comprises *ground*, *laws of metaphysics*, and *grounded*. Note that grounding, as has been said in Sect. 3, depicts the layered structure of the world. We can understand the explanatory inference-rules in an explanatory argument from the ground to the grounded as the *metaphysical laws that govern the layered structure of the world*, the laws (or principles) that govern ground.^34^ The explanatory rules in an explanatory argument from the ground to the grounded allow us to explanatorily reason from the former to the latter. That is to say it is those explanatory rules that tie the grounded to the ground. It is plausible to understand these rules as *metaphysical laws* holding between the ground and the grounded. We can now state what the results of my arguments in Sect. 5 mean outside the framework of explanatory arguments: If unionism is true, a grounding claim *Γ* < *ϕ* is itself explained by appeal to the *metaphysical laws* holding between *Γ* and *ϕ*. The grounding claim that the existence of a complex whole is grounded in the existence of its parts, if true, is itself explained by appeal the metaphysical laws applying to wholes and parts.^35^

From this I conclude that the metaphysical laws that govern the layered structure of the world, the metaphysical principles of grounding (in Schaffer’s words) are the obvious candidates to figure in the metaphysical explanation of grounding facts.^36^ Hence, it follows that on a unionist picture, according to which grounding just is metaphysical explanation, the *grounds* of grounding facts should incorporate the rules or laws that govern the connection between the grounded and the ground.

How does this relate to (GE)? It is plausible that essentialists would want to understand these laws of metaphysics as laws of essence captured by essentialist facts.^37^ Note that according to (GE) grounding facts are themselves grounded in precisely such essentialist facts about the essential connection between (some or all constituents) of the ground and the grounded. We can take Dasgupta’s (2014, p. 566) example from Sect. 7 to illustrate this.

(C): The fact that event *e* contains people engaged in conference-conducive activities (C-activities) grounds the fact that *e* is a conference.

According to Dasgupta’s version of (GE), (C) is itself grounded in the fact that it is *essential* to conferences to contain people engaged in C-activities. The essentialist fact in which (C) is grounded has it that the metaphysical connection between being a conference and containing people engaged in C-activities holds as a matter of essence. Thus, (GE) explains *why* the metaphysical explanatory connection between the ground and the grounded holds. It answers the meta-grounding question by citing a law of essence holding between the ground and the grounded. By appealing to some *rule* or law of essence, (GE) offers a rule-explanation of grounding facts. Thus, in light of my arguments in Sects. 5 and 8, (GE)—contrary to (ZGA)—is still a live option for the unionist.

## The straight forward account (SFA)

There is still one approach to the meta-grounding question left to discuss. Bennett (2011) and deRosset (2013) independently develop an account which Litland (2017) refers to as the “Straight Forward Account” (SFA). According to (SFA), a fact about grounding, like (*Γ * < *ϕ*) is itself grounded in *Γ*.

(SFA): If *Γ * < *ϕ*, then (*Γ* < *ϕ*) is itself grounded in *Γ.*

Due to space limitations, I will confine myself to Bennett’s rationale for this view. Bennett holds that grounding is a “superinternal” relation.

A superinternal relation is one such that the intrinsic nature of only *one* of the relata—or, better, one side of the relation—guarantees not only that the relation holds, but also that the other relatum(a) exists and has the intrinsic nature it does.” (Bennett 2011, p. 32).

Consider physicalism. The following is a very rough formulation of physicalism in terms of grounding:

(P): The physical facts ground the mental facts.

Bennett argues, that physicalists are *not* claiming that the grounding relation between physical and mental facts obtains in virtue of *both sides* of the relation. The physicalists’ claim rather is that it is in virtue of the intrinsic nature of the *physical facts*, the more fundamental facts, that (a) the mental facts, the less fundamental facts, obtain and that (b) the grounding relation between the physical and the mental facts obtains (Bennett 2011, p. 33). Bennett concludes from this that grounding is a *superinternal* relation, that it holds in virtue of *only one* side of the relation. If physicalism is true, if the mental facts are grounded in the physical facts, then by the superinternality of grounding the fact that the mental facts are grounded in the physical facts is itself grounded in the physical facts.

So, Bennett’s rationale for (SFA) is grounding’s superinternality.^38^ One might wonder, however, whether Bennett’s characterization of superinternality appeals to essences. Bennett puts forth that “[a] superinternal relation is one such that the *intrinsic nature* of only *one* of the relata […] guarantees not only that the relation holds, but also that the other relatum(a) exists and has the intrinsic nature it does” (2011, p. 32; *emphasis added*). Should we thus take Bennett to hold a version of (GE), since this seems to entail that the grounding relation between *Γ* and *ϕ*, holds (at least partly) in virtue of the *intrinsic nature*, the *essence*, of *Γ*? No. Bennett is very clear that, on her account, it is not the essences of the physical facts but the physical facts that ground the grounding fact that the mental facts are grounded in the physical facts. So, (SFA) should not be subsumed under (GE).

## (SFA) and unionism are incompatible

Dasgupta (2014) voices a crucial difficulty for (SFA) that is along the same lines as the objection against (ZGA). Consider the following two grounding claims:

(D): (*p *∨ *q*) is grounded in *p.*
(DN): (¬¬*p*) is grounded in *p.*

According to (SFA), both of these grounding facts are themselves grounded in *p*. From his unionist perspective, Dasgupta (2014, pp. 571–573) claims that this renders (SFA) plainly wrong. Intuitively, we expect (D) and (DN) to have different metaphysical explanations. It is plausible that an explanation of why *p* makes (*p *∨ *q*) obtain involves some fact concerning *disjunction*, while an explanation of why *p* makes (¬¬*p*) obtain involves something about *negation*. If grounding just is metaphysical explanation, (D) and (DN) cannot have the same ground—that would entail that they also have the same metaphysical explanation, which appears intuitively incorrect. I think that this criticism has a point. Yet, one has to clearly see that it hinges on the truth of unionism. Thus, while this objection might not show (SFA) to be false, it might still show the account to be incompatible with unionism.^39^ Just like in the case of (ZGA), also here we have an inconsistent triad:

(DE′): (D) and (DN) have different explanations.
(SFA): (D) and (DN) are both grounded in *p.*
(U): Grounding is metaphysical explanation.

The unionist proponent of (SFA) might try to employ Litland’s strategy of rejecting (DE′). It might be argued that it is only in one sense of “explanation” that (D) and (DN) have different explanations. In another sense of “explanation”, (D) and (DN) have the same explanation. In my discussion of (ZGA), I have identified these two senses of “explanation” as input-explanation on the one side and rule-explanation on the other side.

Note, however, that with regard to (SFA) this strategy runs into the same problem as with regard to (ZGA): Again, merely introducing a sense of “explanation” for which (DE′) does not seem plausible does not suffice as a rejection of (DE′). This is because the point of the objection against a unionist version of (SFA) is that it’s the plausibility of (DE′) that casts doubt on any explanation that assigns the same *explanans* to (D) and (DN). The dialectic runs parallel to the discussion of (ZGA). The unionist proponent of (SFA) could even make the Litlandian move to try to explain away the plausibility of (DE′) by distinguishing *different ways* in which a grounding claim like *Γ * < *ϕ* is itself explained by *Γ* (pertaining to the rules or laws involved). Yet, as long as there is no reason to accept this picture other than its compatibility with (SFA), the conjunction of (SFA) and unionism is in an equally weak dialectical position as the conjunction of (ZGA) and unionism.

It is also possible to argue directly against the conjunction of (SFA) and unionism by applying the same argument used in our discussion of (ZGA). As with (ZGA), the success of a unionist version of (SFA) hinges on whether it is plausible that appealing to *Γ* can be seen as a satisfactory metaphysical explanation of *Γ* < *ϕ*. In Sect. 5 I argue that since grounding claims express an explanatory connection between the ground and the grounded and not just any connection, a satisfactory metaphysical explanation of a grounding fact explains *why* there is such an explanatory connection between the ground and the grounded. In the framework of explanatory arguments this amounts to the question as to why the argument from the ground (*Γ*) to the grounded (*ϕ*) is indeed explanatory. Yet, how should we understand this question outside the framework of explanatory arguments? Given unionism, ‘*Γ* < *ϕ*’ amounts to the claim that *Γ* metaphysically explains *ϕ*. The unionist meta-grounding question then asks *why Γ* metaphysically explains *ϕ*. An answer to this question should tell us why *Γ* is explanatorily related to *ϕ*. So, outside the framework of explanatory arguments, the question a satisfactory metaphysical explanation of the grounding claim *Γ* < *ϕ* has to answer is best understood in the following way:

(Q′): Why is *Γ* explanatorily relevant to *ϕ*?^40^

Simply referring to *Γ* does not seem to be an adequate answer to this question.^41^ Take the following example. Suppose the mental fact that I have a headache is grounded in some physical fact *f*. Now consider the following grounding fact (H):

(H): *f* < I have a headache.

The unionist meta-grounding question of why (H) holds, takes the following form: Why is *f* explanatorily relevant to my having a headache? Intuitively, this question cannot be adequately answered by simply referring to *f*.^42^ The question of why *f* is *relevant* in a metaphysical explanation of my having a headache will much rather be answered by a fact about the relation between *f* and my having a headache (or by a fact about the relation of the physical and the mental in general).

In conclusion, if grounding is indeed *superinternal*, *f* might ground the fact that *f* grounds my having a headache. Yet, *f* does not explain why *f* explains my having a headache. I have argued that it is plausible that a metaphysical explanation of (H) should explain the explanatory relation between *f* and my having a headache, just as a metaphysical explanation of (D) should explain the explanatory relation between *p* and (*p *∨ *q*). That is to say on a *unionist* picture, the meta-grounding question regarding (H) is to be understood along the lines of (Q′). It asks *why f* is explanatorily relevant to the mental fact that I have a headache. Since (SFA) cannot answer this *unionist* meta-grounding question, (SFA) and unionism are incompatible. The answer to the unionist meta-grounding question, as argued in Sect. 8, will much rather include some reference to a law holding between the grounded and its ground.

## Conclusion

I have argued that both (ZGA) and (SFA) are incompatible with unionism (Sects. 5 and 10). The argument for this derives its force from the point that a satisfactory metaphysical explanation of why a grounding fact holds explains why there is an explanatory relation among the ground and the grounded. Assuming unionism, the meta-grounding question concerning some grounding claim *Γ* < *ϕ* amounts to asking why *Γ* is explanatorily relevant to *ϕ.* Arguably, neither (ZGA) nor (SFA) are fit to answer this question. Further, I have argued that it is plausible that the unionist meta-grounding question is to be answered by appeal to some rules or laws that govern the connection between the ground and the grounded (Sect. 8). Since (GE)’s answer to the meta-grounding question plausibly appeals to essentialist laws holding between the ground and the grounded, (GE)—contrary to its competitors (ZGA) and (SFA)—is still a live option for the unionist. Hence, if unionism is true, then—in light of the arguments in this paper—(GE) has to be preferred over (ZGA) and (SFA).

Note that *nothing* has been said about whether or not any of the accounts investigated are compatible with *separatism*. Nothing in the arguments in this paper speaks against a separatist version of (ZGA), (SFA), or (GE). What the arguments in this paper establish, however, is that the answer to the question of what grounds grounding in part depends on how we decide the debate between unionism and separatism.

## Footnotes

1 This question is also sometimes called ‘the question of iterated ground’. Hence, answers to it are oftentimes referred to as ‘accounts of iterated ground’.

2 It is Litland (2017) who gives Bennett’s (2011) and deRosset’s (2013) account this label.

3 Note that in a recent manuscript Sider (manuscript) defends an account according to which the answer to the meta-grounding question might be complex. Sider does not give a concrete answer to the meta-grounding question; instead he discusses what kinds of facts can play a role in grounding ground. Even though this approach is very interesting and deserves further discussion, I will not go into it in this paper, since Sider’s account is too general to have commitments concerning the question of unionism versus separatism. Note, however, that a lot of other results of Sider’s work in this manuscript (especially his discussion of what I call (GE) and (SFA)) are of great relevance to this topic.

4 However, due to reasons of space, I will not focus on deRosset’s specific version of (SFA) but rather discuss the incompatibility of (SFA) and unionism in general.

5 But see Jenkins (2008) for a view that “explanation” is semantically general with respect to ontological categories, such that also objects can figure in explanations.

6 Note that in the following I will remain neutral over the question what kind of entities the grounding connective connects. I will sometimes employ “fact-talk”, acting as if grounding is a relation among facts, and sometimes I will treat the grounding idiom as connecting statements. This allows me to depict the different accounts of iterated ground in as unaltered a form as possible. While the official views of the two accounts that I am going to be primarily concerned with, Dasgupta’s and Litland’s, take grounding to be a sentential connective, Dasgupta (2014) employs fact-talk for the sake of simplicity.

7 I do not mean to suggest, however, that (F) is uncontested. See Wilson (2014) for a criticism of the ground-theoretical characterization of fundamentality. Tahko (2018) offers an alternative characterization of fundamentality in terms of ontological minimality.

8 Note that, strictly speaking, it does *not* follow from (Purity) and (F) that *all* grounding facts are themselves grounded. Sider (manuscript, pp. 2, 24) mentions the possibility of “pure”, ungrounded grounding facts. The possibility of pure, ungrounded grounding facts requires two assumptions: First, that there can be non-fundamental facts that only involve fundamental notions; and second, that ‘ground’ itself is to be regarded as a fundamental notion. Even though both of these assumptions can be considered controversial (Sider rejects the second one), they are neither ruled out by (Purity) nor by (F). Note that all I need to carry on with my argument is the claim that grounding facts like (2) are themselves grounded, which is entailed by (F) and (Purity). Nonetheless, we can establish the stronger claim that *all* grounding facts are themselves grounded just by generalizing (Purity) to (Purity*):

(Purity*): No constitutive part of a fundamental fact is non-fundamental.

Note that (Purity*) does not only speak about notions truths or facts involve but about their constitutive parts in general. Facts about grounding are complex in as much as they connect *non-fundamental* facts with (more) fundamental facts. Take e.g. the physicalist’s grounding-claim that the mental fact that I have a headache is grounded in some physical fact *f*. This claim connects the *non-fundamental* (mental) fact that I have a headache to a (more) fundamental (physical) fact *f*. So, even if there might be “pure” grounding facts (not involving non-fundamental notions) there cannot be “pure*” grounding facts, since all grounding facts essentially have a non-fundamental fact as a constitutive part.

9 Note that replacing (Purity) with (Purity*) would yield the even stronger result that *all* grounding facts must be grounded (see fn. 8).

10 There is a debate about whether what we could call the *meta-grounding thesis*, the claim that grounding facts are themselves grounded threatens the intelligibility of the entire notion of ground (cf. Sider 2011). It is feared that the meta-grounding thesis entails infinitely descending, non-terminating dependence chains, such that the reality of any given grounding fact would, as Schaffer puts it, “be infinitely deferred, never achieved” (Schaffer 2010, p. 62). Recent work about well-foundedness and grounding, however, has shown that this worry is unjustified. The meta-grounding thesis gives rise to infinitely many dependence chains, yet it does not entail that any of these are infinitely descending or non-terminating. For a detailed discussion of these points, see Dasgupta (2014), Dixon (2016), Rabin and Rabern (2016) and Sider (manuscript).

11 Note that Litland’s official view is that the grounding operator is flanked by sentential expressions. It takes a plurality of sentences and a single sentence (in that order) to form another sentence which expresses a grounding statement. ‘*Γ* < *ϕ*’ then reads ‘some sentences *Γ* factively ground a sentence *ϕ*’; while ‘*Γ* ⇒ *ϕ*’ reads ‘some sentences *Γ* non-factively ground a sentence *ϕ*’. However, for the sake of simplicity, I will allow myself to alternate between talk about facts being grounded in other facts and talk about sentences being grounded in other sentences (see also fn. 6).

12 This result follows directly from (ZGA) if we—as Litland (2017, p. 285) explicitly does—assume the Cut principle: if *Γ* < *ϕ* and Δ, *ϕ* < *ψ*, then *Γ*, Δ < *ψ*. (Note that Litland’s (2017, p. 285, fn. 16) formulation of the Cut principle includes a typo—his last ‘*ϕ*’ clearly is supposed to be a ‘*ψ*’. This is fixed in the formulation given here.) According to (ZGA), 0 < *ϕ* is fully, factively grounded in (i) 0 and (ii) 0 ⇒ *ϕ*. Further, (ZGA) has it that 0 ⇒ *ϕ* is itself (factively) grounded in 0. Hence, given Cut, we can conclude that 0, 0 < (0 < *ϕ*), which can be simplified to 0 < (0 < *ϕ*). In short, assuming Cut, (ZGA) entails that if some truth *ϕ* is zero-grounded, the truth that *ϕ* is zero-grounded is itself zero-grounded.

13 Note that zero-grounding is *trivially factive*: The factive grounding claim Δ < *ϕ* holds iff (i) each δ ∈ Δ is the case and (ii) the non-factive grounding claim Δ ⇒ *ϕ* holds. When it comes to zero-grounding, (i) is vacuously true: trivially, each δ ∈ Δ is the case, if Δ is the empty set. (see also fn. 18). .

14 His answers to these questions are fairly technical. Since I lack the space to go into the technical details of Litland’s arguments, I will sketch the intuitive idea behind zero-grounding informally. This strategy glosses over some details of Litland’s approach but gets across the core idea.

15 Note that in addition to his official account of zero-grounding in terms of explanatory arguments, Litland also elaborates a metaphor he finds in Fine (2012) to make the idea more intuitive: “Think of a machine generating truths from other truths. The machine is fed truths, churning out truths grounded in the truths it is fed. A truth is *ungrounded* if the machine never churns it out; a truth is *zero-grounded* if the machine churns it out when it is fed no input. […] When the machine is fed no input the machine, instead of remaining idle, ‘simulates’ the results of being fed various input. In simulating what happens when it is fed the propositions Δ the machine proceeds just as it would have if it in fact had been fed Δ as input. If, when running the simulation, the machine churns out *ϕ*, the machine ends the simulation and churns out Δ ⇒ *ϕ*. Since the machine was fed no input this means that Δ ⇒ *ϕ* is zero-grounded if true.” (Litland 2017, p. 287) So, ‘Δ ⇒ *ϕ*’ refers—staying in the metaphor—to the fact that there is a mechanism in the machine that takes Δ and delivers *ϕ*. What grounds the fact that there is such a mechanism in the grounding machine? According to Litland (2017, pp. 288–289), we should understand this fact as zero-grounded.

16 For Litland, the notion of an explanatory inference is primitive. Also, since his primary goal is a formal logic of iterated ground, he does not wish to ultimately settle the material question of what the basic explanatory inferences are. Nevertheless, he names conjunction-introduction, disjunction-introduction and the inference from *a* is *F* to *a* is *G*—where *F* is a determinate of the determinable *G*—as plausible candidates for explanatory inferences (Litland 2017, p. 289).

17 Since Litland takes non-factive ground to be primitive, I take it that he actually derives (GEA) from (GEA_NF_) by adding (i).

18 Note that according to (ZGA), all true, non-factive grounding claims, as well as all true grounding claims involving the zero-ground are themselves *factively* grounded in the zero-ground. So, when it comes to zero-grounding (GEA) applies (as opposed to (GEA_NF_)). Still, we can equate the fact that some *ϕ* is *factively* zero-grounded with the fact that there is an explanatory argument to *ϕ* from the *empty set of premises* without incorporating the factivity condition (i). This is because, when it comes to zero-grounding, (i) is vacuously true: trivially, each δ ∈ Δ is the case, if Δ is the empty set. (see also fn. 13). I am grateful to an anonymous reviewer from *Synthese* for pushing me on this point.

19 This is a version of Dasgupta’s (2014, p. 573) criticism he voices against (SFA). I’ll discuss Dasgupta’s objection to (SFA) in Sect. 10.

20 That is to say that intuitively the explanation of why a conclusion like the trivial cake-truth holds is not exhausted by the premises that lead to this conclusion. It also comprises inference rules which are fundamentally different from premises. For the importance of this distinction see Carroll (1895).

21 Note that, according to (ZGA), both arguments have the same premise set: the empty one.

22 “This is easiest to see by looking at the machine picture. When the machine is asked to simulate being fed *p* it will churn out both *p* ⇒ *p* ∧ *p* and *p* ⇒ *p* ∨ *p*. But there is no reason to think that the simulations the machine runs in order to reach these outputs are the same; in particular, the two simulations might comprise the application of different mechanisms.” (Litland 2017, p. 303).

23 Note that both separatists and unionists would agree on this point.

24 Assume that *q* is entirely unrelated to *p.* Also, note that ‘→’ here just expresses material implication.

25 Litland (2017, p. 297) has it that if Δ ⇒ *ϕ* is the case—if there is an explanatory argument from Δ to *ϕ—*then every δ ∈ Δ is relevant in explaining why *ϕ* is the case. However, *q* is entirely unrelated to *p *→ *p*. Hence the argument from *q* to *p *→ *p*, though classically valid, must be *non-explanatory*.

26 See also fn. 16.

27 We could grant Litland a unionist retreat by conceding that (ZGA) is compatible with the identification of grounding and metaphysical *input*-explanation (as opposed to *rule*-explanation). However, since it is the appeal to *rule*-explanation that answers the relevant question (Q) and that accounts for the relevant intuition (DE), this unionist retreat seems to be a Pyrrhic victory at best. I am indebted to an anonymous reviewer from *Synthese* for this point.

28 Thanks to an anonymous reviewer from *Synthese* for raising this important objection.

29 Remember that in the framework of explanatory arguments, the meta-grounding question of why *p* (non-factively) grounds *p* ∧ *p* boils down to the question of what makes the argument from *p* to *p* ∧ *p* explanatory.

30 Note that in the following discussion of this position I will not differentiate between (GE) and (GE′). Henceforth, ‘(GE)’ rather denotes either of these two formulations. Context will sometimes suggest the one over the other. Still, I take it to be illuminating to mention both these formulations here.

31 Note that Audi (2012a, p. 109) comes close to endorsing a version of (GE) when he argues that “grounding holds only between facts whose constituent properties are essentially connected”. Yet, it is important to note that for Audi grounding facts are *not* themselves *grounded* in essentialist facts about the essential connectedness of the ground and the grounded. In other words, the relation between the essentialist fact and the grounding fact is not itself grounding. Audi (2012b, p. 695) ultimately argues that “[…] we should not expect every grounding fact to be grounded, even in a fact about essential connection”. This is why he falls short of being a proponent of (GE), at least in the way I have introduced (GE) in this paper.

32 I do not wish to hide that despite its initial plausibility, (GE) has its problems. One critical question concerns the ground of the essentialist fact in which the grounding fact is partially grounded. This question is crucial since the way we answer it might decide whether the account gives rise to an infinitely descending chain of grounds. Due to lack of space, I cannot discuss these issues here. I will, however, point to Dasgupta’s answer to this problem. Dasgupta’s account avoids such non-terminating chains by claiming that essentialist facts are not apt for being grounded in the first place. For a detailed discussion of Dasgupta’s version of (GE), which he calls “brute essentialism”, see Dasgupta (2014, 2016). For criticism, see e.g. deRosset (2013) and Sider (manuscript).

33 This is in line with Sider’s (manuscript) verdict that one would expect *Γ* < *ϕ* to have a ground that connects the facts *Γ* and *ϕ*.

34 Note that while Schaffer casts the link-component in metaphysical explanations, i.e. the metaphysical laws, as “metaphysical principles of grounding” in his (2017a, p. 3), his introduction of metaphysical laws in his (2017b) is more ecumenical since it does not assume that laws of metaphysics are cast in terms of grounding. For the purposes of this paper it makes sense to think about metaphysical laws as metaphysical principles of grounding, yet, as Schaffer (2017b) points out, it is not necessary to do so.

35 Note that it is not necessary to make the strong claim here, according to which it is the metaphysical laws *alone* that do the explanatory work. What is claimed, however, is that metaphysical laws must figure in the *explanantia* of grounding facts for they do some explanatory work. This is in line with my use of ‘rule-explanation’ in Sect. 5.

36 Analogous arguments for the logical and causal cases can be had: An explanation of the fact that some conclusion *ϕ* is logically entailed by a premise-set *Γ* should answer the question of *why* this entailment relation holds. Hence, it is plausible that the inference-rules on the basis of which *ϕ* follows from *Γ* should figure in the *explanans* of the fact that *Γ* logically entails *ϕ*. Equivalently, an explanation of the fact that *ϕ* is caused by some event(s) *Γ* should answer the question of *why* this causal relation holds. We would thus also expect the laws of nature responsible for this causal relation to figure in the *explanans* of the fact that *Γ* causes *ϕ*. So, the result that the metaphysical laws play a role in the explanation of grounding facts generalizes: Assuming that logical, causal, and metaphysical explanations all have a tripartite structure of source, link, and result, any explanation of why such an explanation holds, has to talk about the link-component of the respective explanation.

37 Note that it is neither ubiquitous among essentialists to understand essence claims in terms of the laws of metaphysics, nor is it necessary to cast laws of metaphysics in essentialist terms. Dasgupta (2014, p. 568), e.g., concedes that metaphysical laws need not be cashed out in terms of essence. For a *constructional account* of metaphysical laws and their role in metaphysical explanation, see Wilsch (2016). The claim here is just that if there is reason to speak of some metaphysical laws or principles that hold between the grounded and its ground, it is very plausible that the essentialist would regard them as laws of essence governing the *essential connection* that holds between (some or all constituents of) the grounded and its ground.

38 But see Carnino (2017) for a view that grounding is not superinternal.

39 Sider (manuscript) has an argument against (SFA) that does not seem to depend on the truth of unionism. If successful, his point casts doubt on both a unionist and a separatist version of (SFA). He begins by noting that grounding either *is* metaphysical explanation (unionism) or a quasi-causal fact backing metaphysical explanation (separatism). From this he concludes that the grounds of grounding facts ought to be analogous to the grounds of explanatory or causal facts. But then, even on the separatist version, (SFA) is implausible, since, whatever the grounds of the fact that *c* causes *e* might be, it is plausible that they won’t just encompass *c.*

40 Note that this is supported by Litland’s own interpretation of his framework of explanatory arguments: Litland (2017, p. 297) has it that “if Δ ⇒ *ϕ* is the case then […] every δ ∈ Δ is relevant in explaining why *ϕ* is the case”. In other words, if there is an explanatory argument from some premise set (Δ) to a conclusion (*ϕ*), then every member of Δ is explanatorily relevant to *ϕ*. It is, hence, plausible that a satisfactory metaphysical explanation of why Δ ⇒ *ϕ* holds has to answer the question of why every δ ∈ Δ is explanatorily relevant to *ϕ*.

41 Audi (2012b, p. 695) seems to agree: “Suppose we said that [x is maroon] grounds [x is red] just in virtue of the fact that *x* is maroon. Plainly this would fail to explain *why* [*x* is maroon] grounds [*x* is red].”

42 Sider (manuscript, pp. 6–7), who raises a similar objection, agrees that *f* does not contain anything *relevant* to the relation of ground.
